# Lack of pain relief during labor is blamable for the increase in the women demands towards cesarean delivery: a cross-sectional study

**Published:** 2017-12

**Authors:** OM Shaaban, AM Abbas, RA Mohamed, HAA Hafiz

**Affiliations:** Department of Obstetrics & Gynecology, Faculty of Medicine. Assiut University; Technical Institute of Nursing. Assiut University, Egypt; Department of Obstetrics & Gynecological Nursing. Faculty of Nursing. Assiut University, Egypt

**Keywords:** Knowledge, pain relief, labor analgesia, vaginal delivery, cesarean section

## Abstract

**Background:**

This study aims to assess knowledge, attitude and acceptance of antenatal women for pain relief methods during labor and to know the effect of presumed availability of pain relief methods during labor on the attitude of women towards the mode of delivery.

**Materials and Methods:**

A cross sectional study was conducted at a tertiary hospital between January and December 2016. A structured interview questionnaire had been administered including data related to current pregnancy, knowledge, attitude and previous experience of pain, labor analgesia, women’s attitude toward the mode of delivery and its relation to the availability of adequate analgesia during labor. Visual analog scale (VAS) was used to assess pregnant women’s attitude towards pain in general and that related to the process of labor (past and expected experience).

**Results:**

Eight hundred and fourteen women were included in the study. The majority of our participants (82.9%) were unaware about the availability of labor analgesia. Sixty of the study participants preferred cesarean section (CS) to avoid labor pain. Availability of adequate pain relief during labor could decrease the women decision of CS by more than 50% in women expecting moderate and severe pain during labor compared to non-availability of pain relief (9.6% vs. 22.7% and 8.2% vs. 28.1% respectively).

**Conclusion:**

There is a great lack of knowledge regarding the availability of pain relief during labor. Lack of pain relief during labor can be responsible for more than half of women’s intention to prefer CS as a mode of delivery.

## Introduction

Natural labor and its pain is probably the most painful event in many women’s life. Consequently the majority of women today require some form of analgesia during labor. Different methods of labor analgesia have reported over the years, in spite of that, pain relief in labor is still controversial (Mugambe et al., 2007). The lack of awareness, acceptability and availability of effective labor analgesia in developing countries is the major obstacle regarding its routine use (Naithani et al., 2011).

Labor is considered a physiological process best managed with the least interference as possible (Caton et al., 2002). Pain relief is an effective and helpful way of reducing the amount of pain associated with uterine contractions. This may involve the use of pharmacological or non-pharmacological techniques or a combination of these methods (James et al., 2012, Madden et al., 2013).

Pharmacological methods commonly used for labor analgesia include: Inhalations with Nitrous Oxide gas, opioid or narcotics drugs such as morphine through different injection routes and local nerve block techniques during labor frequently used to numb nerves in the vaginal area. However, these techniques are not always effective. Regional analgesia/anesthesia is the most effective analgesia involving techniques that block pain nerves from the uterus and birth canal with the use of local anesthetics. This includes epidural, spinal and combined spinal and epidural (CSE) techniques. Nevertheless, this effective option is not always available or acceptable for the patients in the developing countries (Jones et al., 2012).

Cesarean section (CS) is one of the most frequently performed surgical procedures worldwide (Arjun, 2008). There is a trend of an increasing CS rate in most developed and developing countries and this constitutes a major public health problem because CS increases the health risk for mothers and babies compared with normal deliveries (Betran et al., 2016).

The reasons for this increase are multi-factorial including medical, cultural, economic, legal, psychological, socio-demographic factors or non-medical reasons such as fear of labor pain or inadequate pain relief techniques during labor (Linton et al., 2004, Zwecker et al., 2011, Mi et al., 2014).

In Assiut Women Health Hospital, the biggest tertiary hospital in Upper Egypt, the most recent clinical audit showed that CS rate was 32% in 2008 and 36% in 2011 (Abdel-Aleem et al., 2013). Increasing rates of birth by CS are an issue of concern among public health officials and the medical community in many countries. Knowledge about the real causes of the increase in CS rate in our country is lacking. Furthermore, we are unaware how far the fear of pain and lack of pain relief during labor affects a woman’s request to CS.

The current study aims to assess knowledge, attitude and acceptance of antenatal women for pain relief during their upcoming labor and to evaluate the effect of presumed availability of pain relief on antenatal women’s decisions about the mode of delivery.

## Materials and methods

### Study type, setting and duration

The study was a prospective cross-sectional study carried out from the first of January 2016 until the 31st of December 2016 in the Antenatal care Clinic of Assiut Women Health Hospital, Egypt. The study protocol was approved by the Assiut Medical School Ethical Review Board. The non- interventional nature of the study and respect of patients’ confidentiality were clear to the patient and patient’s oral consent to participate had been obtained before starting the interview.

### Study population

The study included all consecutive pregnant women presenting to the clinic in their third trimester of pregnancy (> 28 weeks). We included women with no medical or obstetric complications during pregnancy that necessitate absolute indication of CS. Women who had a history of any chronic medical diseases, those scheduled for elective CS and those refused to participate were excluded from the study.

### Sample size

Sample size calculation was based on the primary outcome (the effect of availability of efficient pain relief during labor on the percentage of women who opt for CS on demand). Previous studies in the same setting showed that the percentage of women who choose CS on demand was 11.5 % of all CS (Abdel- Aleem et al., 2013). Presuming availability of pain relief during labor can decrease CS on demand by 50% a sample size calculation was performed. Using two sided chi-square (χ2) test with α of 0.05 and 80% power to detect 50% reduction in CS on demand in case of availability and non-availability of pain relief during labor, a minimum sample size of at least 814 women was calculated (taking a ratio of 1:1 unexposed (no pain relief) to exposed (availability of pain relief), [Odds ratio of 0.47] (Epi-infoTM, CDC, USA, 2008).

### Study tools

Recruitment included all consecutive eligible participants until the required sample size had been fulfilled. The study tool was a structured interview questionnaire introduced by a trained nurse at the time of admission to the study. The questionnaire included questions related to personal (including detailed contact details), obstetric history and current pregnancy data. Furthermore, the questioner asked questions of women’s knowledge about labor analgesia, attitude towards dealing with labor pain, knowledge about different methods of labor analgesia, source of information about labor analgesia, knowledge about who is providing labor analgesia.

Additionally, we also collected information about women’s wish to have labor analgesia in the upcoming delivery, the preferred method of labor analgesia and if refused the reasons for this refusal. More importantly, it included questions about women’s preferred mode of delivery. Women were asked about their choice between vaginal delivery and CS if there would be no pain relief during labor and in case of its availability.

For pain scoring the standard 10 cm visual analog scale (VAS) was explained to the participants (Bouhassira et al., 2005). We used the VAS score to assess pregnant women’s attitude towards pain (past experience of severe pain, previous and expected labor pain). The severity of pain was assessed with VAS (with 0 = no pain, grades 1-3 means mild pain, grades 4-6 means moderate pain and grades 7-10 means severe pain).

### Study outcomes

The primary outcome of the study was the effect of supposed availability of efficient pain relief during labor on the percentage of women who opt for CS on demand.

### Statistical analysis

The collected data were coded, tabulated and analyzed using the statistical package for social science programs (SPSS) Chicago, IL, USA, version 21. Continuous data were expressed as frequency, percentage, means and standard deviation. Discrete data were expressed as frequency and percentage. Comparison between groups was done using Student’s T-test. Level of significance “P” value was evaluated, where P value <0.05 was considered statistically significant.

## Results

One thousand and twenty-three women were approached to participate in this study. Two hundred and nine women were excluded due to presence of different exclusion criteria (111 women were scheduled for elective CS, 71 women decline participation in the study and 27 women had reported medical complications during pregnancy). The remaining 814 were included in the final analysis.

The mean age of study participants was 26.05 ± 5.39 years, and the mean parity was 2.39 ± 1.54. Baseline characteristics were listed in Table I. Concerning knowledge about labor analgesia, Table II shows that the majority of the participants (82.9%) never received information about labor analgesia. 93.5% of the women who had some knowledge reported that it should be provided through injections. Nearly one third (30.9%) pointed that the provider of labor analgesia should be the obstetrician, while 12.9% reported that the anesthesiologist in charge was the one who should be responsible for analgesia during labor. Regarding the source of information about labor analgesia, we observed that 71.2% of the study group obtained their knowledge from friends and relatives, while 19.4% obtained information from audiovisual media.

**Table I:** Demographic characteristics of the study participants.

Characteristics	Study participants (n= 814)	
	n	%
Age, mean ± SD	26.05 ± 5.39	
Parity, mean ± SD	2.39 ± 1.54	
Level of education		
Illiterate	299	36.7
Read & write	41	5.1
Primary education	145	17.8
Secondary education	246	30.2
University education	83	10.2
Occupation		
Working	27	3.3
Not working	787	96.7
Residence		
Rural	480	58.9
Urban	334	41.1
Previous deliveries		
Vaginal delivery	464	57
Cesarean delivery	202	24.8
Both	83	10.2

**Table II:** Distribution of participants on the basis of knowledge, methods and provider of labor analgesia.

Items of questionnaire	Study participants	
	n	%
Knowledge about labor analgesia *		
Yes	139	17.1
No	675	82.9
Source of information (n=139)		
Friends and relatives	99	71.2
Media	27	19.4
Printed literature	5	3.6
Previous labor	5	3.6
Others	3	2.2
Methods of labor analgesia (n=139)		
Parenteral injections	130	93.5
Lower back injection	6	4.3
Don't know	3	2.2
Provider of labor analgesia (n=139)		
Obstetrician	43	30.9
Anesthesiologist	18	12.9
Nurse	36	25.9
Don't know	42	30.2

*(n=814)

Table III shows participants’ attitude towards labor analgesia. It was found that the majority (80.4%) of the studied group had experienced moderate or severe pain during their previous deliveries. As regard to expected labor pain, 75.7% of the women had expected that their labor pain would be severe this time. By shedding light on the participant’s opinion regarding labor analgesia, 11.9% of the study group refused receiving any forms of analgesia during labor of their upcoming delivery.

**Table III:** Participants’ attitude towards labor analgesia.

Items of questionnaire	Study participants (n=814)	
	n	%
Past experience of labor pains		
No pain	141	17.3
Mild pain	19	2.3
Moderate pain	242	29.7
Severe pain	412	50.7
Expected labor pains		
No pain	85	10.4
Mild pain	16	2.0
Moderate pain	97	11.9
Severe pain	616	75.7
Participants’ opinion regarding labor analgesia		
Preferred	717	88.1
Not preferred	97	11.9
Reason for refusal of labor analgesia *		
Want to experience labor pains	55	56.7
The methods don’t work	19	19.6
The methods may be harmful to the fetus	17	17.5
Others	6	6.2

When labor analgesia was presumed to be unavailable at the time of upcoming labor, 629 women (77.3%) of the study participants opted to give birth vaginaly and 185 (22.7%) women preferred CS. On the other hand, when the availability of labor analgesia was supposed, the vast majority of the study participants 741 (91%) opted to give birth normally and only 9% opted to have CS, this difference was statistically significant (p = 0.001) (Table IV).

**Table IV:** Women’s choice of mode of delivery in case of presumed availability and non- availability of pain relief during labor.

	Non- availability of pain relief	Availability of pain relief	P-value
Prefer VD	629 (77.3%)	741 (91.0%)	0.001*
Prefer CS	185 (22.7%)	73 (9.0%)	0.001*

VD = vaginal delivery, CS = cesarean section, * Significant difference

Table V illustrates the effect of the participant’s expected labor pain on their choice of mode of delivery. It was noticed that the higher the degree of women expectation of labor pain, the more the tendency toward choosing CS in the upcoming delivery and this significantly correlated with the availability of effective pain relief during labor. Furthermore, 28.1% of the women who were expecting severe pain during their upcoming delivery preferred CS in case of non-availability of pain relief as compared to 8.1% if pain relief was presumed to be available (p = 0.001).

**Table V:** The effect of the participant’s expected labor pain on their choice of mode of delivery in case of presumed availability and non-availability of pain relief during labor.

	VD			CS		
	Availability of pain relief	Non-availability of pain relief	P-value	Availability of pain relief	Non-availability of pain relief	P-value
	n (%)	n (%)		n (%)	n (%)	
Expected labor pain			0.001*			0.001*
No pain	29 (3.9%)	5 (0.8%)		56 (76.7%)	80 (43.2%)	
Mild pain	12 (1.6%)	5 (0.8%)		4 (5.5%)	11 (6%)	
Moderate pain	90 (12.2%)	55 (8.7%)		7 (9.6%)	42 (22.7%)	
Severe pain	610 (82.3%)	564 (89.7%)		6 (8.2%)	52 (28.1%)	
Total	741 (100%)	629 (100%)		73 (100%)	185 (100%)	

VD = vaginal delivery, CS = cesarean section, * Significant difference

## Discussion

It is considered that an interview is a superior method for in-depth data collection because a relationship develops with the subjects in the study and the researcher feels confident that they would respond openly and honestly, thus enhancing the quality of the data (Englander, 2012). Thus, we conducted a study on 814 pregnant women who were in the third trimester pregnancy and eligible for normal delivery in our hospital to (1) assess their knowledge, attitude and acceptance for pain relief methods during labor and (2) to know the effect of pain relief during their upcoming delivery on their decisions about the mode of delivery. We found that there is a great lack of knowledge regarding pain relief during labor including needs, providers and methods. Furthermore, the availability of effective labor analgesia may contribute in decreasing CS by more than 50%, particular those women expecting moderate to severe pain during their upcoming delivery as compared to supposed non-availability.

In the present study, the participants mentioned that the main reason for their preference of CS was labor pain. Our results are similar to the results obtained in another study conducted in the same setting by Abdel-Aleem et al. who found that a substantial part of the increased CS rate was probably CS on demand, mainly based on fear of pain during labor (Abdel-Aleem et al., 2013).

Pain relief in labor had become one of the essential aspects in the management of childbirth and there are an increasing number of women worldwide who are using analgesia during labor. In this study we observed that the majority of women had no or poor knowledge about labor analgesia (82.9%). This poor knowledge of women is probably due to lack of antenatal education and counseling about labor analgesia in our hospital. Availability of good information is lacking due to the high workload and shortage of nurses. Above this there is a lack of epidural analgesia provision at our facility as in most of the general hospitals in our region.

In an Indian study Shidhaye et al. (2012) also revealed that almost the entire study group (98.48%) irrespective of age, educational level and socioeconomic status didn’t have any information about labor analgesia. Similarly, Barakzai et al. (2010) in Pakistan performed a study among 131 women to assess awareness of women regarding analgesia during labor and found that less than half of the women were aware of labor pain relief methods.

Moreover, the results of our study show that the main source of information for the study participants were friends or relatives. This can be attributed to our cultural practices as the majority of the women feel comfortable to discuss personal matters with friends while doctors have a strong influence over decision making in health seeking practices. The previous finding agreed with the study results of Mung’ayi et al. in Kenya which showed that friends, the antenatal clinic and books/leaflets were the major source of information about pain relief methods (Mung’ayi et al., 2008). In India James et al., (2012) found that most of the women (78%) had heard about methods to relieve labor pain mainly through the media and through their doctors.

The main reason to perform this study was to know the attitude of women towards pain relief. Our results revealed that the past experiences of labor pain were graded as severe as 10 and the expected labor pain were graded as severe as 8-9. In South Africa Ibach et al. (2007) conducted a study among 30 healthy women who presented for antenatal care, they also found that the past experience and expected labor pain were graded as severe as 8.4 and many women were scared of labor pain. In contrast Naithani et al. (2011) reported that the past experience and expected labor pains were graded as mild or moderate as 3 or 4 and most of these women were confident that they could cope with the labor pains in this Indian study.

Concerning participants’ opinion regarding labor analgesia, the results of the present study revealed that the majority of women preferred pain relief during labor. The same finding was reached by Olayemi et al. who found that about two thirds of the respondents in a Nigerian study were willing to accept analgesia if offered (Olayemi et al., 2003). On the other side, the most common reason for refusal in our study was the desire of antenatal woman to experience normal delivery. This can be attributed to traditional values in which pain in labor is considered a positive feature of labor and the idea of relieving it is often opposed. Similarly, Mugambe et al. (2007) found that most of the women believed that they have to experience pain during labor.

The other goal of this study was to investigate the effect of presumed availability of pain relief on the antenatal women’s decisions about the mode of delivery. When the option of availability of labor analgesia was offered, the vast majority of the studied women opted for a normal vaginal delivery and slightly less than one tenth opted to give birth by CS, a difference which is statistically significant (p = 0.001). Furthermore our results indicate that there is a highly statistically significant relation between the desire of vaginal or cesarean delivery and their expected labor pain (p = 0.001) in case of non-availability of pain relief and availability of pain relief. In a previous study Abdel-Aleem et al. (2013) also reported that making labor painless or increase the availability of pain relief during labor may decrease CS on demand subsequently decreasing the CS rate.

Finally, we believe that our study represents a comprehensive attempt to document the effect of the participant’s labor pain on their choice of mode of delivery in case of presumed availability and non- availability of pain relief during labor. As with any research, however, there are limitations to this study that should be considered when interpreting our results. The results of this study in Assiut City may not generalize because the study sample is small. In addition the study was conducted at a single setting, thus limiting the reliability of the data which could have been gained from basing it in multi-setting.

In conclusion, in many resource-poor countries there is a lack of knowledge regarding the need for pain relief during labor, the various methods of labor pain relief , their advantages and disadvantages. There is a statistically significant relation between participant’s labor pain and desire of vaginal or cesarean delivery in case of presumed availability and non-availability of pain relief during labor. Antenatal women should be educated and counseled about the need for labor analgesia and the available options by the provision of information leaflets, labor pain websites and childbirth preparation classes.
